# Brain effect mechanism of lever positioning manipulation on LDH analgesia based on multimodal MRI: a study protocol

**DOI:** 10.1186/s12906-024-04549-4

**Published:** 2024-06-24

**Authors:** Xing-chen Zhou, Long-hao Chen, Shuang Wu, Kai-zheng Wang, Zi-cheng Wei, Tao Li, Yuan-shen Huang, Zi-han Hua, Qiong Xia, Zhi-zhen Lv, Li-jiang Lv

**Affiliations:** 1 The Third Affiliated Hospital of Zhejiang, University of Traditional Chinese Medicine, Hangzhou, Zhejiang China; 2 The Third School of Clinical Medicine, Zhejiang Chinese Medical University, Hangzhou, Zhejiang China; 3 Research Institute of Tuina (Spinal Disease), Zhejiang Chinese Medical University, Hangzhou, Zhejiang China

**Keywords:** Lumbar disc herniation, Multimodal magnetic resonance imaging, Lever positioning manipulation, Analgesia mechanism, Brain

## Abstract

**Introduction:**

The clinical symptoms of Lumbar Disc Herniation (LDH) can be effectively ameliorated through Lever Positioning Manipulation (LPM), which is closely linked to the brain's pain-regulating mechanisms. Magnetic Resonance Imaging (MRI) offers an objective and visual means to study how the brain orchestrates the characteristics of analgesic effects. From the perspective of multimodal MRI, we applied functional MRI (fMRI) and Magnetic Resonance Spectrum (MRS) techniques to comprehensively evaluate the characteristics of the effects of LPM on the brain region of LDH from the aspects of brain structure, brain function and brain metabolism. This multimodal MRI technique provides a biological basis for the clinical application of LPM in LDH.

**Methods and analysis:**

A total of 60 LDH patients and 30 healthy controls, matched by gender, age, and years of education, will be enrolled in this study. The LDH patients will be divided into two groups (Group 1, *n* = 30; Group 2, *n* = 30) using a random number table method. Group 1 will receive LPM treatment once every two days, for a total of 12 times over 4 weeks. Group 2 will receive sham LPM treatment during the same period as Group 1. All 30 healthy controls will be divided into Group 3. Multimodal MRI will be performed on Group 1 and Group 2 at three time points (TPs): before LPM (TP1), after one LPM session (TP2), and after a full course of LPM treatment. The healthy controls (Group 3) will not undergo LPM and will be subject to only a single multimodal MRI scan. Participants in both Group 1 and Group 2 will be required to complete clinical questionnaires. These assessments will focus on pain intensity and functional disorders, using the Visual Analog Scale (VAS) and the Japanese Orthopaedic Association (JOA) scoring systems, respectively.

**Discussion:**

The purpose of this study is to investigate the multimodal brain response characteristics of LDH patients after treatment with LPM, with the goal of providing a biological basis for clinical applications.

**Trial registration number:**

https://clinicaltrials.gov/ct2/show/NCT05613179, identifier: NCT05613179.

## Introduction

Lumbar disc herniation (LDH) primarily manifests as lower back and leg pain [1, 2]. This condition is distinguished by its high incidence, significant disability rate, and the absence of specific pharmaceutical remedies, thereby imposing a substantial socioeconomic burden on society [3–5]. In a comprehensive global disease survey encompassing 291 conditions, LDH holds the highest rank in terms of disability, with a prevalence rate of 9.4% [6]. Furthermore, it exhibits a noticeable trend towards affecting a younger demographic, with an escalating risk of disability as age advances [7]. Surgical treatment is a commonly employed clinical approach, yet concerns arise due to its recurrence rate and potential complications. Surveys indicate that 2.7% of individuals experience postoperative complications [8], with a 5-year reoperation rate reaching 11% [9, 10]. In contrast, non-surgical treatments are garnering increasing support from both medical practitioners and patients.

Lever positioning manipulation (LPM) is a supplementary therapeutic method for Lumbar Disc Herniation (LDH) and is extensively utilized in China [11, 12]. This technique is generally performed by skilled medical professionals who exert a controlled force on the patient's lower back muscles and spine to mitigate pain. However, the fundamental mechanisms underlying the pain relief provided by LPM, as well as the neurophysiological regulation of LPM by the brain, are not yet fully understood. Recent research postulates that pain relief results from the profound integration of incoming signals within the brain [13–16]. Consequently, the core mechanisms by which LPM alleviates pain in LDH necessitate a profound exploration into how the brain orchestrates pain sensitivity.

Magnetic resonance imaging (MRI) is a radiation-free technology characterized by exceptional spatial and temporal resolution, enabling non-invasive examination of the human brain [17–19]. This approach allows for the direct study of the human body, circumventing the limitations associated with reliance on animal models. In recent years, the investigation of the mechanisms by which the brain exerts analgesic effects through MRI has emerged as the cutting-edge and focal point of this discipline.

Functional magnetic resonance imaging (fMRI) stands as the most extensively employed technique within the realm of MRI, notable for its heightened sensitivity in scrutinizing the cerebral functional attributes. A multitude of studies have consistently emphasized that the brain's functional alterations take precedence over structural changes [19, 20]. At present, prevalent data analysis methods for resting-state fMRI encompass the examination of metrics such as mean amplitude of low-frequency fluctuation (mALFF) [21], regional homogeneity (ReHo) [22], and functional connectivity (FC) [23]. Moreover, magnetic resonance spectroscopy (MRS) represents an additional technical modality within the MRI domain, being the sole non-invasive means for quantitatively detecting alterations in cerebral substances [24, 25]. MRS can discern a plethora of compounds within the brain, including N-acetylaspartate (NAA), choline (Cho), creatine (Cr), glutamate (Glu), glutamine (Gln), gamma-aminobutyric acid (GABA), and glucose, among others. Consequently, MRS serves as a supplementary avenue of research to fMRI, facilitating a more profound exploration of metabolic changes within the implicated brain regions.

This study employs multimodal MRI techniques, specifically ALFF and ReHo, to analyze crucial brain regions associated with analgesic effects. Subsequently, statistically significant differences in brain regions are extracted as regions of interest (ROI) for the analysis of brain network FC. This analysis is aimed at monitoring neurochemical changes related to MRS within the ROI brain regions, with the overarching goal of investigating the neuroimaging mechanisms behind the analgesic efficacy of LPM in the context of LDH.

## Materials and methods

### Study design

**Fig. 1:**
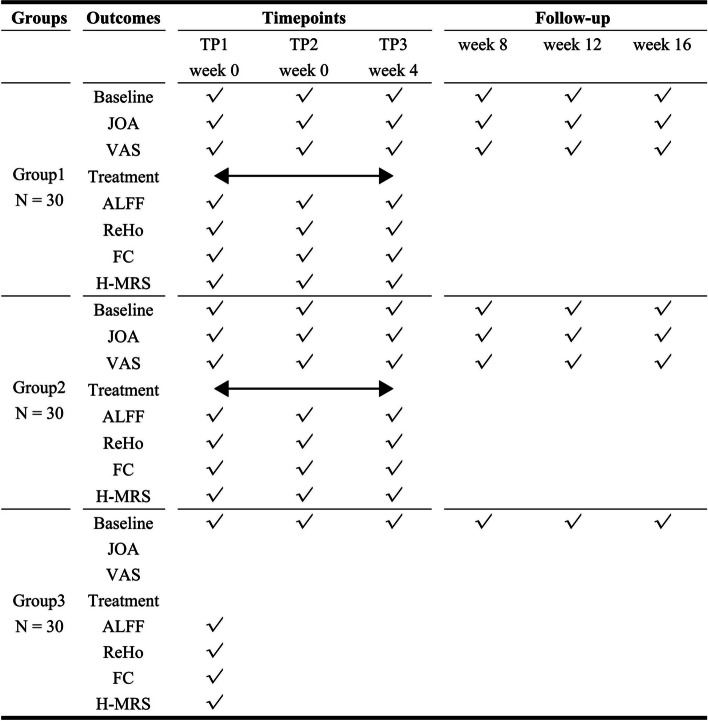
Standard protocol items: recommendations for interventional trials (SPIRIT) schedule of the trial

This non-randomized clinical trial is set to enroll 60 patients diagnosed with Lumbar Disc Herniation (LDH) and 30 healthy controls (HCs). Fig. 1 illustrates the schematic outline of the study protocol. The research protocol has received approval from the Institutional Review Board (No. ZSLL-KY-2022–049-01) at the Third Affiliated Hospital of Zhejiang Chinese Medical University, P. R. China. Additionally, this protocol is registered on ClinicalTrials.gov (registration No. NCT 05613179). Written informed consent for participation will be obtained from all participants involved in this study.

### Participants

Sixty patients with LDH and thirty with HCs will be recruited for this study. All 60 LDH patients were randomly divided into 2 groups with 30 patients in each group. The Group1 will be treated with LPM and Group2 will be treated with sham LPM.

Inclusion criteria for the LDH patients are as follows: participants must meet the diagnostic criteria for lumbar disc herniation, including protrusion types such as central, paracentral, far lateral, foraminal, and subarticular, with imaging findings that align with neurological localization. The age range for participants is 18 to 65 years, encompassing any gender, and they must be right-handed. Participants should be in the non-acute phase of the condition, experiencing mild to moderate pain and functional impairment, with the duration of symptoms exceeding 2 weeks. They should have a Visual Analog Scale (VAS) score greater than 4 and Japanese Orthopaedic Association (JOA) scores less than 15 [26]. Eligible participants must not have taken analgesics, neurotropic nutrition drugs, or sedatives in the past month and should not have undergone systemic treatment. Additionally, they should not have received spinal manipulation or other physical therapies in the past month. Finally, participants must voluntarily agree to participate in this study and have signed an informed consent form.

Exclusion criteria for the study are as follows: (1) Patients with concurrent internal medicine or gynecological conditions known to cause lower back pain, such as nephritis, urinary stones, gynecological inflammations, and uterine abnormalities. (2) Individuals with severe primary diseases impacting the cardiovascular, cerebrovascular, liver, or kidney systems. (3) Those with neurogenic functional disorders, psychiatric conditions, a history of significant head trauma, or a history of unconsciousness. (4) Individuals diagnosed with primary sciatica or dry sciatica. (5) Those with lumbar spondylolisthesis. (6) Patients suffering from lumbar tumors or tuberculosis. (7) Individuals with severe osteoporosis or localized skin lesions in the lumbar area. (8) Patients experiencing painful conditions beyond the lumbar region. (9) Those with diseases characterized by structural changes in the brain. (10) Individuals with impaired consciousness, severe visual or hearing impairments, speech disorders, or others who are unable to complete health assessments. (11) Individuals with dental implants, metal stents, or other elements that may compromise MRI imaging. (12) Those with a fear of MRI or other reasons that prevent undergoing MRI scans. (13) Patients diagnosed with lumbar disc herniation but who are asymptomatic.

We recruited age- and gender-matched healthy controls with no history of Lumbar Disc Herniation (LDH) (Group 3) through poster advertisements. The eligibility criteria for these participants were as follows: they must be right-handed, aged between 18 and 65 years, and of any gender. These individuals should have no history of lower back pain and must not have received any medication or undergone relevant physical therapy in the past month. Additionally, they should have a clear understanding of the research process and express willingness to participate by signing the informed consent form.

### Participant recruitment

All patients with LDH and HCs will be recruited from both outpatient and inpatient services of the Third Affiliated Hospital of Zhejiang Chinese Medical University, P. R. China, as well as from the community through advertising campaigns. The recruitment period is set from September 2021 to September 2025.

### Randomization, allocation concealment, and blinding

After baseline assessments, eligible participants with LDH will be randomly allocated to either the LPM group or the sham-LPM group through a randomization process. A random number grouping table will be generated based on the predetermined number of cases and a specified random proportion. These randomization tables will be created using SAS 9.0 software and will be managed by independent research assistants who are not involved in the participant recruitment, assessment, or intervention processes.

Independent research assistants will inform eligible participants of their group assignment results via phone communication. Due to the nature of the interventions, it is not possible to implement blinding for researchers, participants, and practitioners throughout the course of the experiment. However, laboratory technicians and researchers responsible for conducting statistical analyses will be kept blinded to group assignments to ensure the objectivity and integrity of the data analysis.

### Procedure

Multimodal fMRI and MRS scans were conducted for patients with LDH in Group 1 and Group 2 at three distinct time points (TPs): before LPM (time point 1, TP1), within one hour after the first intervention (Group 1 receiving LPM and Group 2 receiving sham LPM) (time point 2, TP2), and within one hour after the last intervention (Group 1 with LPM and Group 2 with sham LPM) (time point 3, TP3).

Patients in Group 1 and Group 2 were additionally required to fill out clinical questionnaires before undergoing the multimodal fMRI and MRS scans at two time points. They were instructed to rate the severity of their LDH symptoms using the VAS, where 0 represents 'no pain' and 10 indicates the 'strongest imaginable pain', and to complete the JOA assessment forms. The differences in VAS and JOA scores between these two time points were calculated to assess changes in pain and functional status.

For the healthy controls in Group 3, only a single session of multimodal fMRI and MRS scan was performed, and they did not participate in completing any clinical questionnaires.

#### Lever Positioning Manipulation (LPM)

**Fig. 2:**
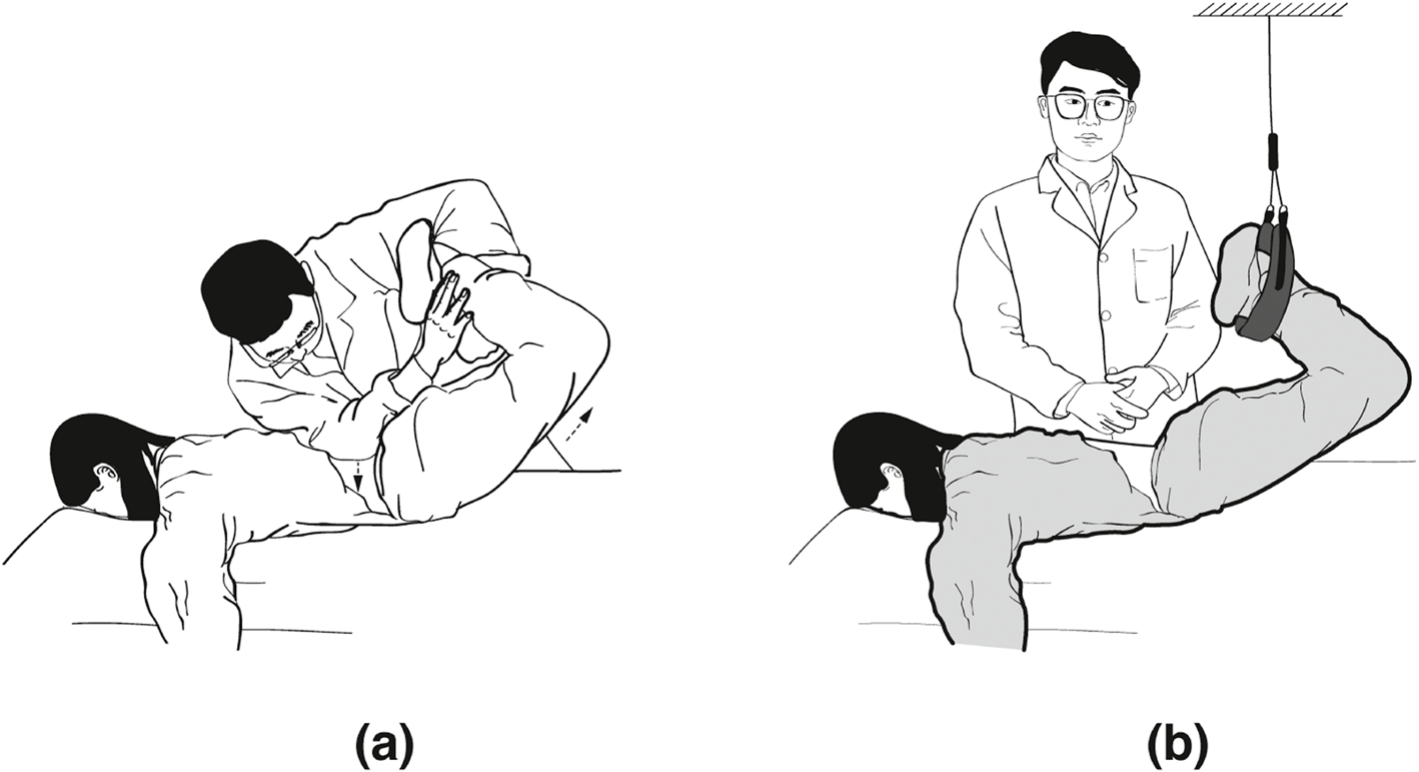
Lever positioning manipulation (LPM) operation diagram: **A** Massaging and manipulation for lumbar muscular relaxation. **B** LPM

LPM Treatment [26]: All LPM treatments were conducted by the same rehabilitation expert, LLJ, who has 33 years of experience in the field. The specific LPM procedure entailed positioning the patient with flexed knees and hips, crossing the lower limbs. The practitioner pinpointed the site of the lumbar disc herniation, using their right elbow to stabilize at this point while holding the patient's ankles with both hands. Utilizing the principle of leveraged force, the practitioner lifted and pulled the patient’s lower limbs upwards and inwards to just before the point of hyperextension. Upon reaching the trigger point, the practitioner executed a rapid pull with the lever, feeling for a 'click' or loosening at the herniation site, at an angle of approximately 5°. During this lever maneuvering and pulling, the patient was instructed to exhale (Fig. 2).

Sham-LPM Treatment: All preparatory steps, patient positioning, and the duration before the commencement of Sham-LPM were identical to those of the LPM treatment, thus effectively minimizing operational bias. The key difference in sham-LPM is that when the patient's position reaches the critical point, the operator does not perform a swift twisting motion. Instead, a protective belt is used to maintain the patient’s position unchanged (Fig. 2).

#### Multimodal fMRI and MRS Scan

Participants in this study were recruited and evaluated at the Massage Department of the Third Affiliated Hospital of Zhejiang Chinese Medical University. Those who met the inclusion criteria underwent data collection using a Siemens Skyra 3.0 T superconducting MRI scanner at Zhejiang Hospital (MAGNETOM Skyra 3.0 T, Siemens, Germany). To minimize confounding factors, clinical interventions for patients with LDH were consistently performed at Zhejiang Hospital, with MRI data collection taking place within 1 h of completing the intervention.

For the LDH group, multimodal fMRI and MRS scans were conducted both before and after immediate treatment. Conversely, the healthy control group underwent a single session of multimodal fMRI and MRS scanning at the time of their enrollment. It is common for patients to experience more pronounced pain sensations on the day of their medical consultation. Hence, to ensure the timely acquisition and reliability of brain function data in response to pain stimuli, MRI scans were conducted on all study participants on the same day they were enrolled and had completed their baseline data assessment.

For structural imaging, a Gradient echo sequence 3D-T1 will be utilized with the following parameters: 249 slices, repetition time (TR) of 2530 ms, echo time (TE) of 2.89 ms, slice thickness of 1.2 mm, layer spacing of 1.0 mm, voxel size of 1 mm × 1 mm × 1 mm, flip angle of 9°, field of view (FOV) of 256 mm × 256 mm, and a data matrix of 64 × 64. The duration of the 3D-T1 sequence will be approximately 5 min and 53 s.

FMRI scans will be acquired using an echo-planar imaging sequence with these settings: 43 slices, TR of 2680 ms, TE of 30 ms, slice thickness of 3.0 mm, no gap, voxel size of 3 mm × 3 mm × 3 mm, flip angle of 9°, FOV of 192 mm × 192 mm, and a data matrix of 64 × 64. The functional sequence will take about 10 min and 53 s.

MRS scans will be conducted using a PRESS (Point-Resolved Spectroscopy) sequence with these parameters: TR of 1700 ms, TE of 35 ms, slice thickness of 2.0 mm, no gap, voxel size of 10 mm × 10 mm × 10 mm, FOV of 240 mm × 240 mm, and a data matrix of 128 × 128. The PRESS sequence will take approximately 9 min and 13 s.

#### Multimodal fMRI and MRS processing

In this study, we enhanced and streamlined data preprocessing using the Graph Theoretical Network Analysis Toolbox (GRETNA-master; [ https://www.nitrc.org/frs/downloadlink.php/10441](https://www.nitrc.org/frs/downloadlink.php/10441.)) within Matlab 2013b (Mathworks, Natick, MA, USA). The first step involved converting raw fMRI data into a format compatible with both GRETNA and SPM12 (SPM12; [http://www.fil.ion.ucl.ac.uk/spm/software/spm12/](http://www.fil.ion.ucl.ac.uk/spm/software/spm12/)). To ensure signal consistency and ease participant adaptation, the first ten temporal points of the data were excluded.

Subsequently, we carried out a detailed temporal correction to synchronize the time series across all slices. This was followed by a precise correction for head movement, using a six-parameter rigid-body transformation to counteract any participant movement during the scan.

For image registration, resting-state fMRI (rs-fMRI) scans were co-registered with high-resolution anatomical 3D-T1 scans. These scans were then spatially normalized to align with the Montreal Neurological Institute (MNI) template. This step involved using Diffeomorphic Anatomical Registration Through Exponentiated Lie Algebra (DARTEL) for advanced non-linear warping of the 3D-T1 images, and then applying the same normalization parameters to the rs-fMRI scans.

Spatial smoothing was performed by applying a Gaussian kernel with a 3 mm full-width at half maximum to the normalized scans, reducing spatial noise and enhancing signal quality. We ensured that spatial movements and rotations were restricted to less than 1.5 mm and 1.5° in any direction to maintain data integrity.

Lastly, the data underwent detrending and filtering. Detrending, aimed at removing noise factors such as scanner heating, was conducted using linear regression. Filtering was implemented because the Blood Oxygenation Level-dependent (BOLD) signal, generated by spontaneous neuronal activity in the resting state, is predominantly concentrated in the low-frequency range. Thus, high-frequency signals like those from breathing and heartbeats were filtered out, while also avoiding low-frequency drift. The frequency band used in this study was 0.01–0.08 Hz.

#### Calculation of fMRI and MRS

FMRI images, prior to filtering, were transformed into the frequency domain using a fast Fourier transform to obtain the power spectrum. The square root of the power spectrum was averaged across the 0.01–0.08 Hz frequency range at each voxel, and these average values were recorded as the ALFF. To standardize the ALFF for subsequent statistical analysis, the ALFF of each voxel was divided by the global mean ALFF value.

For the calculation of ReHo, data without smoothing were utilized. ReHo was computed through Kendall’s Coefficient of Concordance (KCC) to assess the synchronization of the time series of a given voxel with its 26 nearest neighboring voxels. Individual ReHo maps were then standardized by dividing them by the global mean ReHo value and subsequently smoothed with a Gaussian kernel of 6-mm full width at half maximum.

Brain regions showing statistically significant differences in ALFF and ReHo values were identified as the ROIs for further analysis of brain network FC. FC provides a macroscopic view of the connectivity within the entire brain's functional network and is valuable for identifying neuroimaging markers for LDH.

The results of each independent component in the experiment included a correlated activation time series and an independent 3D spatial distribution of brain voxel images. For this analysis, we used the Group ICA of fMRI Toolbox (GIFT) software (version 3.0, [http://mialab.mm.org/software/gift/](http://mialab.mm.org/software/gift/)) to perform spatial independent component analysis. The component data of the participants were estimated using the algorithm of maximum entropy. The ICASSO method ([http://research.ics.tkk.fi/ica/icasso](http://research.ics.tkk.fi/ica/icasso)), with 30 repeated computations, facilitated the clustering analysis of the final component results, thereby determining the reliability of the component estimation.

^1^H-MRS is used to measure the concentrations of NAA, Cho, Cr and phosphocreatine, and to estimate the concentrations of neurotransmitters such as glutamic acid, glutamine and GABA in the brain. ^31^P-MRS was used to detect adenosine triphosphate, phosphodiester, phosphomonoester, phosphocreatine, and inorganic phosphoric acid, and to estimate brain pH. MR Analysis software was used to automatically analyze the spectral signals and measure the metabolites. MRS Mainly focused on hydrogen proton (^1^H) and phosphorus (^31^P).

#### Image viewing

For the visualization of statistical results, we will utilize the xjview 95 toolkit ([https://www.alivelearn.net/xjview/xjview-9-5-released/](https://www.alivelearn.net/xjview/xjview-9-5-released/)), which will employ the Montreal Neurological Institute (MNI) system for coordinate localization and the Anatomical Automatic Labeling (AAL) 90 atlas for brain region labeling. To create a three-dimensional map of the entire brain, the BrainNet Viewer toolkit will be employed.

Brain regions that will display statistically significant differences will be identified based on specific criteria: a cluster volume threshold (cluster) of ≥ 50 voxels and a single voxel threshold of *P* < 0.005. Additionally, to minimize the likelihood of Type I errors, a False Discovery Rate (FDR) correction (Glickman et al., 2014) will be applied with a threshold of *P* < 0.05. These stringent criteria and visualization tools will facilitate the precise identification and graphical representation of areas of the brain significantly impacted in the study.

### Patient safety

Patient safety measures will include the routine examination of blood, urine, and stools; blood biochemical testing (including liver and kidney function); and electrocardiography, which will be performed for each participant before trial enrollment. Any adverse events caused by LPM will be reported to the project leader, the research institute, and the ethics committee within 24 h and will be documented in the case report form (CRF).

## Discussion

The clinical effectiveness and safety of LPM intervention for LDH have been validated on multiple occasions [11, 12], yet the mechanisms underlying its analgesic effects remain to be elucidated. Most researchers concur that the brain plays a central role in these analgesic effects [13–16]. Multimodal fMRI and MRS techniques allow for a non-invasive direct study of the mechanisms of analgesic effects in the human brain from both qualitative functional and quantitative metabolic dimensions. Numerous studies have demonstrated that combining these two techniques enhances the credibility of research outcomes [27–33].

In our preliminary experiments [26], we observed extensive and immediate activation of brain regions following LPM intervention for LDH, suggesting a central and networked regulation. To further explore the regulatory mechanisms of key brain regions responsible for the analgesic effects and to identify the critical neurotransmitters involved, this study employs multimodal fMRI and MRS techniques. It will analyze brain functional metrics such as ALFF, ReHo, and FC, as well as detect signals related to brain metabolites and neurotransmitters like NAA, Cho, and GABA. The ultimate goal is to elucidate the core neurobiological mechanisms by which LPM intervention for LDH exerts its clinical analgesic brain effects.

## Data Availability

No datasets were generated or analysed during the current study.

## Abbreviations

LDH: Lumbar disc herniation
LPM: Lever positioning manipulation
fMRI: Functional magnetic resonance imaging
MRS: Magnetic resonance spectroscopy
ALFF: Amplitude of low-frequency fluctuation
ReHo: Regional homogeneity
NAA: N-acetylaspartate
Cho: Choline
Cr: Creatine
Glu: Glutamate
Gln: Glutamine
GABA: Gamma-aminobutyric acid
ROI: Regions of interest
JOA: Japanese Orthopaedic Association
VAS: Visual analog scale
KCC: Kendall's coefficient of concordance
FDR: False discovery rate
HCs: Healthy controls
TPs: Time points
MNI: Montreal Neurological Institute
TR: Repetition time
TE: Echo time
FOV: Field of view
CRF: Case report form
